# Aural myiasis in a neonate in peninsular Malaysia

**DOI:** 10.1186/1756-3305-2-63

**Published:** 2009-12-15

**Authors:** Nazni Wasi Ahmad, Anuar Ismail, John Jeffery, Sa'diyah Ibrahim, Azahari Abdul Hadi, Mohd Noor Ibrahim, Heo Chong Chin, Lee Han Lim

**Affiliations:** 1 Medical Entomology Unit, Infectious Diseases Research Centre, Institute For Medical Research, Jalan Pahang 50588, Kuala Lumpur, Malaysia; 2 Kedah State Vector Control Program, 1474, Taman Uda, Jalan Sultanah 05350, Alor Setar, Kedah, Malaysia; 3 Department of Parasitology, Faculty of Medicine, Universiti Malaya, 50600 Kuala Lumpur, Malaysia; 4 Faculty of Medicine, Universiti Teknologi MARA, 40450 Shah Alam, Selangor, Malaysia

## Abstract

Myiasis is a pathological condition in humans and animals caused by various species of dipterous larvae. Myiasis which occurs in a newborn baby is referred as neonatal myiasis. It is a rare condition and there are only a few reports to date. A case of neonatal aural myiasis in a two day old infant is reported in this paper.

## Findings

The first documented case of human myiasis in Malaysia was by Reid [1] of a male who had an ulcer on his big toe infested with the green bottle, *Chrysomya bezziana*. A review on all human cases of myiasis in Malaysia up to 1984 were classified according to Zumpt's nomenclature [2,3]. Only a few cases of myiasis have been reported in Malaysia but this could be due to a lack of documentation. In Malaysia, thus far, only 3 cases of aural myiasis have been described [4-6]. In this paper we report the fourth case of aural myiasis in a 2 day old infant in Malaysia [7].

A two day old male infant from Alor Setar, State of Kedah, was discharged from a private specialist medical centre and brought back home. The child was observed to cry often and the parents subsequently brought him to the hospital for medical examination. It was discovered that the left ear was inflamed. On close examination, the medical officer found moving objects in the ear. The objects were carefully removed using fine forceps and were confirmed to be maggots. No damage to the ear drum was observed. After removing the maggots and administration of appropriate medication, the child was discharged. Four maggots in 70% alcohol were sent to the Medical Entomology Unit, Institute for Medical Research, Kuala Lumpur. The specimens were processed for study and identification according to standard procedures [8]. The specimens were examined under a microscope at 400× magnification.

**Figure 1:**
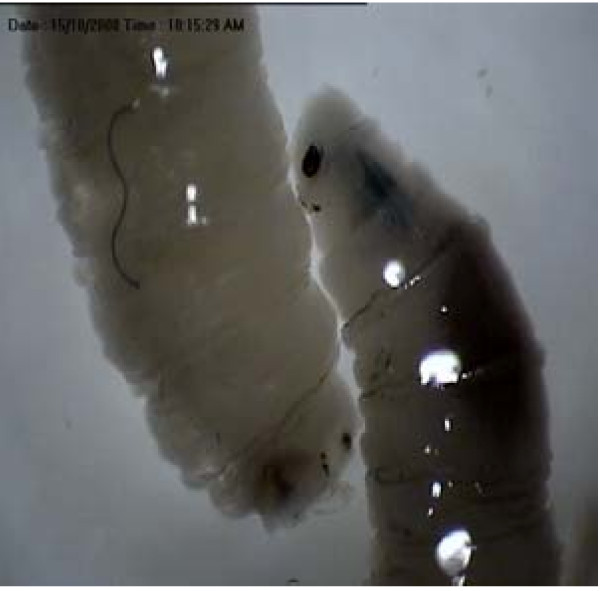
**Posterior spiracle (PS) within a depression/pit of Sarcophagidae**.

**Figure 2:**
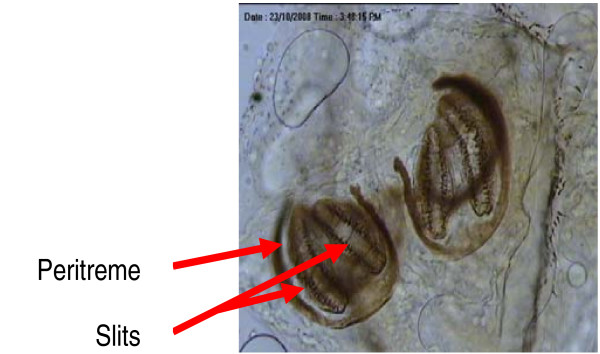
**Spiracles of a second stage larvae showing 2 slits and incomplete peritreme directed away from opening**.

**Figure 3:**
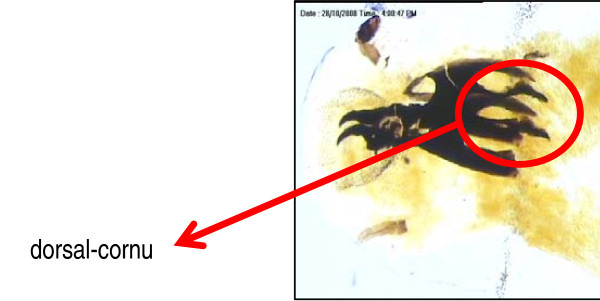
**Picture showing cephalopharyngeal armature with split dorsal cornu**.

Based on the presence of two respiratory slits each in the posterior spiracles, the 4 maggots were identified as second stage maggot of the fly family Sarcophagidae. An important gross taxonomic feature in this family is the nature of the posterior spiracle which is located within a depression/pit at the posterior end of the maggot (Figure 1). Additionally, the maggots were easily recognized as a species of Sarcophagidae by the following features: (i) by the possession of thin incomplete peritreme with respiratory slits directed away from the opening (Figure 2), and (ii) the cephalopharyngeal armature having split dorsal cornu (Figure 3). Flies of this family are larviparous in habit i.e. laying first stage larvae instead of eggs. The mean body length of these larvae was 3.0 ± 0.1 mm indicating that the minimum age of this larvae at the time of removal was at least 12-23 hours old. No attempt was made to identify the specimens to species level as males are needed for this purpose. The adults of these flies can be considered as facultative myiasis producers which opportunistically infest the wounds or body cavities of the host. Human cases of facultative myiasis caused by members of this family Sarcophagidae (Diptera) have been found throughout the world [2,9-11]

Occurrence of infestation may be due to a gravid female being attracted to odour emanating from the ear. The presence of these larvae could also indicate that the neonate probably acquired the infestation while in the hospital prior to being discharged. The first case of auricular myiasis in Malaysia was from a ten year old Indian girl infested with third instar larvae of *Chrysomya megacephala *[4]. The second case was a 41 year old Malay woman from a lower socio-economic environment. The maggots were third instar larvae of the fly *Chrysomya bezziana *[5], while in the third case *Chrysomya bezziana *larvae were also recovered from a 41 year old aborigine male [6]. Aural myiasis in adults infested by Sarcophagidae (Diptera) have been reported in other parts of the world [12,13]. This is the first report of an infant being infested with a species of Sarcophagidae in Malaysia. Myiasis patients should be treated immediately by removing the maggots from the infested area. Although aural and ocular myiasis can be dangerous, they can be treated effectively in neonates with topical and systemic therapies [14].

## Competing interests

The authors declare that they have no competing interests.

## Authors' contributions

NWA, AI, JJ and LHL conceived the paper and wrote the manuscript SI, AAH, MNI and HCC assisted in laboratory studies.
